# Adult T-Cell Lymphoma (ATL) With Erythroderma in Indolent Human T-Lymphotropic Virus Type I (HTLV-1) Infection

**DOI:** 10.7759/cureus.41264

**Published:** 2023-07-01

**Authors:** Abeer Qasim, Sachin Bhandari, Venkata Sri Ramani Peesapati, Harsh R Parikh, Aam Baqui

**Affiliations:** 1 Internal Medicine, BronxCare Health System, Bronx, USA; 2 Internal Medicine, St. George's University School of Medicine, St. George's, GRD; 3 Pathology, BronxCare Health System, Bronx, USA

**Keywords:** diagnosis and treatment of htlv-1 infection, mature t-cell malignancy, erythroderma associated with adult t-cell lymphoma, rare lymphoproliferative neoplasm, adult t-cell lymphoma/leukemia (atl)

## Abstract

Adult T-cell lymphoma (ATL) is a hematological malignancy of CD4+ mature T-lymphocytes commonly associated with chronic human T-lymphotropic virus type I (HTLV-1) infection. Chronic HTLV-1 infection induces oncogenic mutations in CD4+ T-cells, leading to an acute malignant transformation of host cells. Atypically, ATL presents with dermatological and pulmonary symptoms consistent with a “smoldering” disease pattern. We present a case of a 78-year-old male patient with chronic generalized malaise, progressively worsening shortness of breath, and diffuse erythroderma, who was diagnosed with ATL secondary to chronic indolent HTLV-1 infection. We evaluate the multisystemic clinical signs associated with ATL, the comprehensive clinical investigations required to reach a conclusive diagnosis, and the options for long-term clinical management.

## Introduction

Human T-lymphotropic virus type I (HTLV-1) is an RNA virus of the Retroviradae family, Deltaretrovirus genus, that causes an aggressive lymphoproliferative malignancy [1]. In the United States, the occurrence rate of adult T-cell lymphoma (ATL) is roughly 0.05 cases per 100,000 individuals. However, in areas where HTLV-1 is widespread, such as certain regions in Japan, the reported incidence can reach as high as 27 cases per 100,000 people. There is no significant gender bias, and the average age of patients diagnosed with ATL is 62 years. ATL is a manifestation of atypical T-lymphocytes infected with HTLV-1. The clinical features of acute ATL include generalized lymphadenopathy, hepatosplenomegaly, immunosuppression, recurrent infections, lytic bone lesions, persistent hypercalcemia, chronic cutaneous lichenification, and skin lesions [2,3]. Clinical evidence includes complete blood count with significant lymphocytosis, elevated inflammatory markers, elevated lactate dehydrogenase (LDH), and a high CD4:CD8 ratio [4,5]. The smoldering pattern of ATL includes cutaneous manifestations with erythroderma and pulmonary symptoms such as dyspnea, accessory muscle use, and lung lesions visible on chest CT [5]. This case report evaluates a unique case of ATL secondary to chronic undiagnosed HTLV-1 infection. The disease pattern and clinical signs were consistent with the smoldering variant of ATL. 

## Case presentation

A 78-year-old male from Peru presented to the Emergency Department (ED) with complaints of progressively worsening shortness of breath and chronic generalized malaise. The patient also complained of polyuria, intermittent low-grade fevers, and diffuse pruritic skin rashes over the scalp, face, trunk, and extremities. He had a past medical history that includes a history of eczema, liver disease, and bilateral hearing deficits. He traveled from Peru to the United States to visit his family. The patient denied any history of surgeries, allergies, and toxic habits. 

Upon arrival at the ED, the patient was vitally stable with a blood pressure of 140/81 mm of Hg, a heart rate of 84 beats per minute, a temperature of 97.8 degrees Fahrenheit, and a respiratory rate of 17. Skin examination revealed diffuse erythematous skin lichenification, exfoliative white scales, and cracking fissures, but no overt signs of cellulitis. Additionally, there was generalized lymphadenopathy including discrete, non-tender lymph nodes in the pre-auricular, retro-auricular, submandibular, submental, axillary, anterior and posterior cervical, and inguinal regions. The laboratory results showed an increase in white blood cells with a significant rise in eosinophils, as well as elevated levels of LDH and C-reactive protein (CRP) (Table 1).

**Table 1:** Labs at the time of admission. WBC: White blood cell; RBC: red blood cell; MCV: mean corpuscular volume; LDH: lactate dehydrogenase; CRP: C-reactive protein

Labs	Result	Normal Ranges
WBC Count	18.1(H)	4.8-10.8 k/ul
RBC Count	3.40(L)	4.50-5.90 MIL/ul
HGB	9.4(L)	12.0-16.0 g/dl
Hematocrit, Whole Blood	28.8(L)	42.0-51.0 %
MCV	84.7	80.0-96.0 fL
Eosinophil %	22.6(H)	<=5.0- %
Eosinophil Count	4.10(H)	0.05-0.25 k/ul
Calcium, Total Serum	7.7(L)	8.5-10.5 mg/dL
LDH	563 U/L	110/220Units/L
CRP	26.45	<5mg/dl
HTLV LINE IMMUNOASSAY(LIA)	Positive	Negative
Antibody Assay,HTLV 1 and 2	Positive	Negative
IgA	442mg/dl	70-320mg/dl
IgE	7322kU/L	<114kU/L
IgG	4285mg/dl	600-1540mg/dl
IgM	46md/dl	50-300mg/dl
Serum Immunofixation	Lambda Bands	Negative
Leukemia/Lymphoma Evaluation	T Lymphocytes with markedly elevated CD4:CD8 ratio of 21:1	Negative

Per initial findings, the patient was admitted to the hospital for suspicion of chronic infection.   

On admission, the patient received an extensive infectious and immunological workup. The infectious workup was negative for hepatitis C virus, human immunodeficiency virus (HIV), and Strongyloidiasis. However, titers for HTLV-1 antibodies were positive. Quantitative immunoglobulin assay revealed elevated IgG, IgE, and IgA immunoglobulins. Urine immunofixation revealed albumin only. Follow-up serum immunofixation demonstrated increased lambda banding.

**Figure 1:**
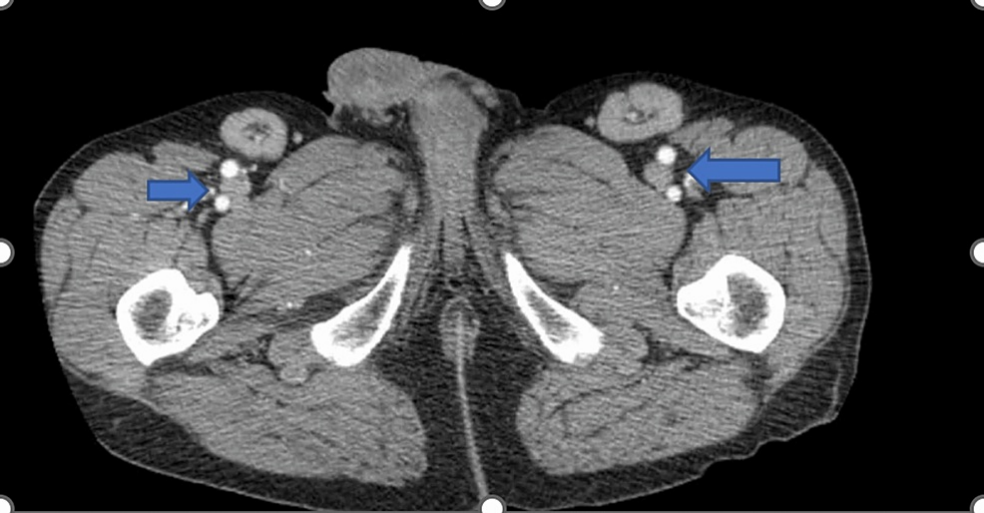
CT chest: bilateral axillary lymphadenopathy (as shown by arrows).

**Figure 2:**
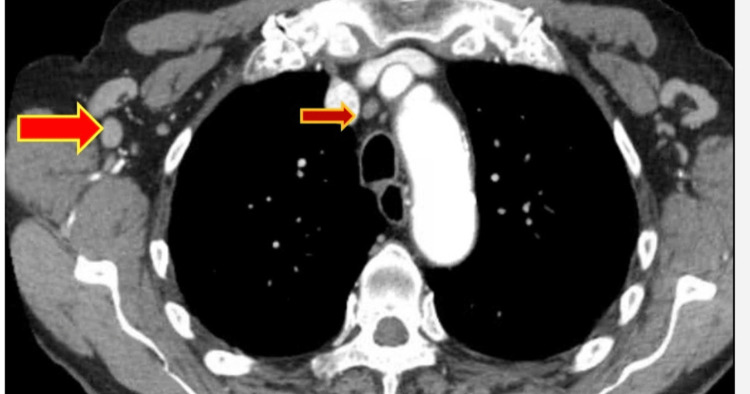
CT abdomen showing inguinal lymphadenopathy.

CT chest and abdomen revealed bilateral axillary and inguinal lymphadenopathy without hepatosplenomegaly (Figures 1, 2).

**Figure 3:**
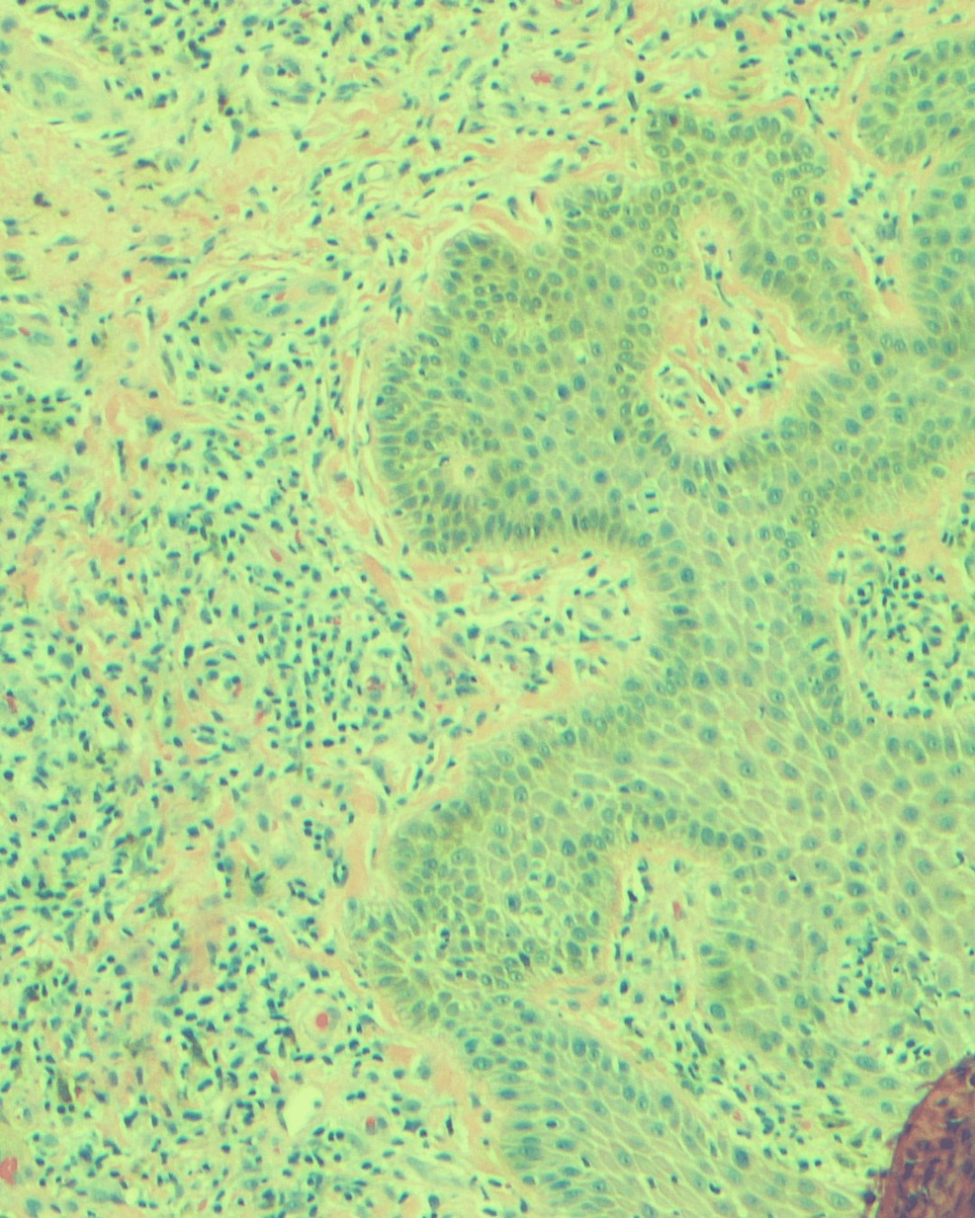
Left thigh skin biopsy: molluscum contagiosum with adjacent lichenoid and interface dermatitis.

This constellation of symptoms directed clinical investigations toward a differential diagnosis for hematological malignancy. T-Lymphocyte flow cytometry on peripheral blood identified a CD4:CD8 ratio of 21:1, without loss of pan T-cell antigens, increasing suspicion for a T-lymphoproliferative malignancy. Initial skin biopsy revealed admixed epidermal monoclonal T-lymphocytes with reactive T-lymphocytes (Figure 3).

**Figure 4:**
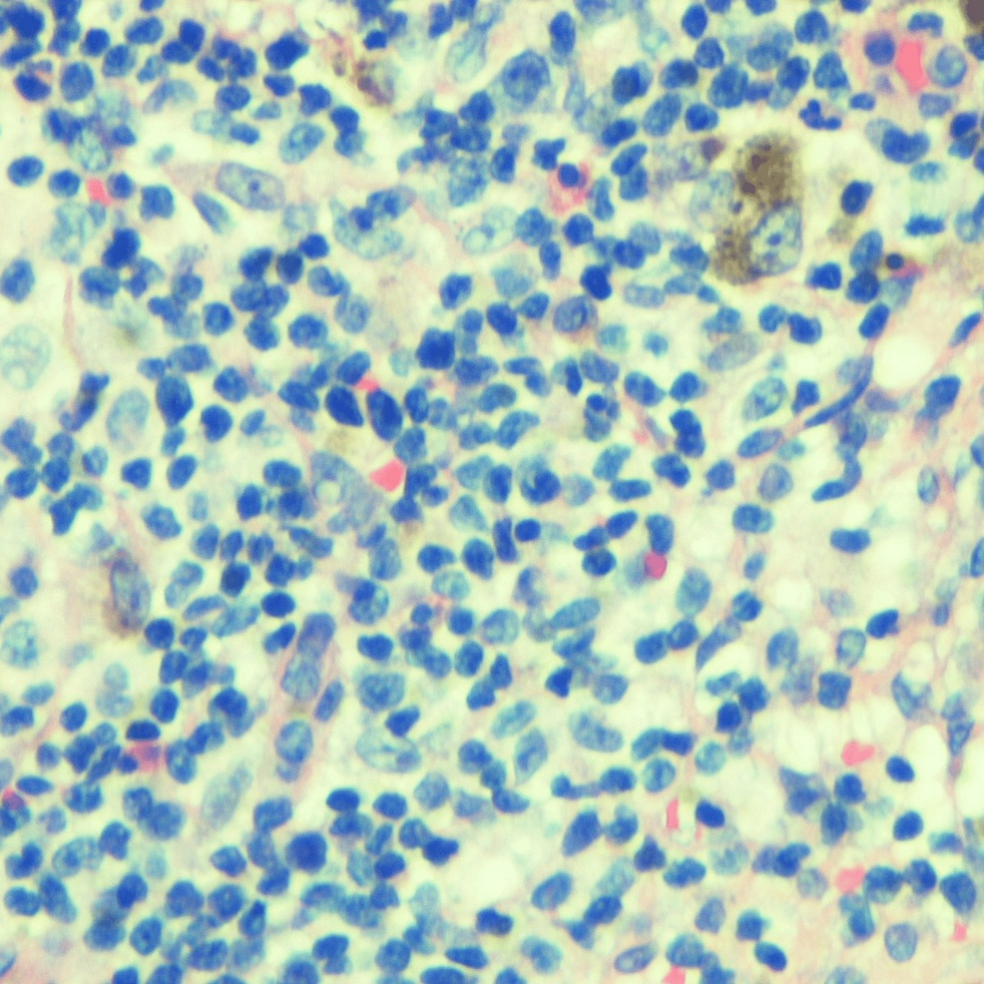
Left axillary lymph node biopsy: mildly altered nodular architecture. Nodular paracortical hyperplasia and melanin-laden macrophages are seen.

**Figure 5:**
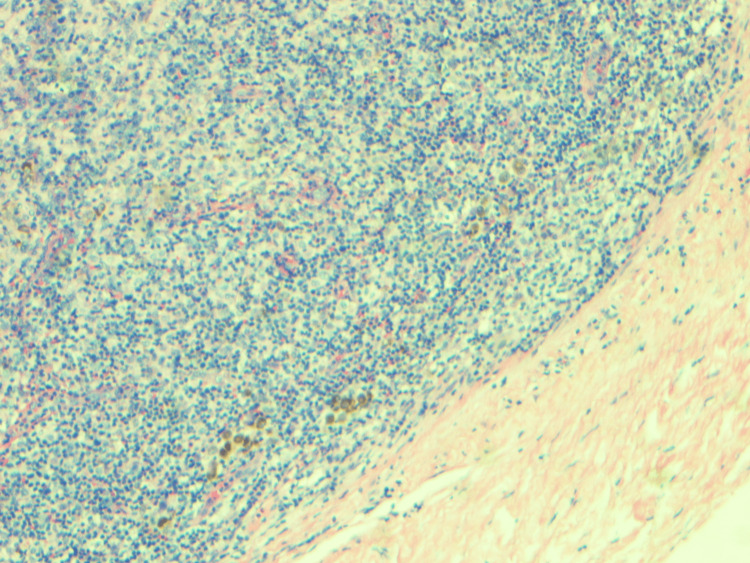
Left axillary lymph node biopsy: the paracortex is expanded, and a polymorphous infiltrate comprising small to intermediate and frequent clusters of large atypical lymphocytes is seen admixed with histiocytes and dendritic cells. Small lymphoid follicles are seen at the periphery. The presence of atypical lymphocytes within the paracortex suggests lymph node involvement by lymphoma.

Excisional biopsy of the axillary lymph node showed atypical lymphocytosis in the paracortex and plasmacytosis within the medullary cords (Figures 4, 5).

Consequent immunohistochemical staining demonstrated lymphocytes positive for CD2+, CD3+, CD4+, CD5+, and CD30+. Immunological and pathological findings concluded a final diagnosis of HTLV-1 ATL with erythroderma.

**Figure 6:**
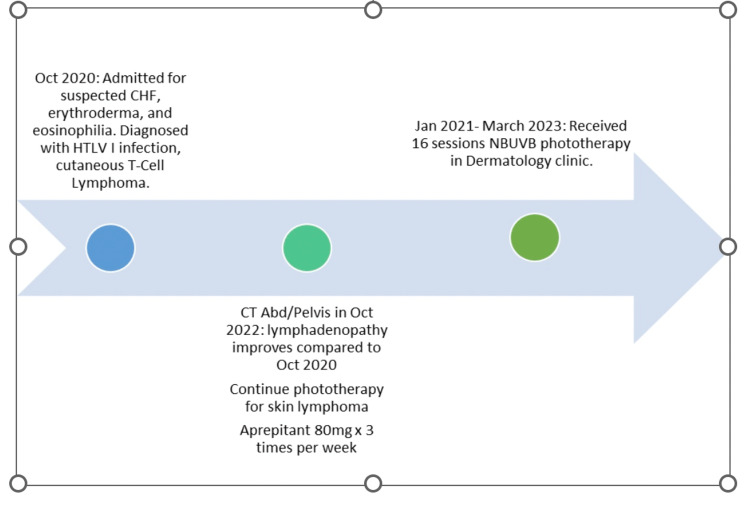
Patient course over the past two years (created by Dr. Sri Ramini Peesapati). HTLV I: Human T-lymphotropic virus type I; NBUVB: narrowband ultraviolet B

In summary, our patient experienced a 12-day hospital course, receiving a final diagnosis of ATL with erythroderma. For management, the patient was given 0.1% triamcinolone topical ointment and scheduled for regular outpatient narrowband ultraviolet B (NBUVB) phototherapy with mogamulizumab adjunctive therapy. Following 12 months of treatment, the patient has demonstrated significant clinical improvement: reduced lymphadenopathy, normalized lymphocyte count, and improvement in skin rash with reduced pruritus. Figure 6 shows the patient's course over the past two years.

## Discussion

HTLV-1 is a pro-oncogenic delta virus that causes a rare form of non-Hodgkin lymphoma/leukemia called ATL. ATL is classified as a lymphoproliferative disorder that usually affects CD4+ and CD 25+ T-cells; however, CD8+ T- cell lymphoma has also been described in the literature [6,7]. HTLV-1 virus has a long latency period, and patients develop ATL in the fourth to fifth decade. The virus is endemic to southwestern Japan, the Caribbean Islands, Africa, the Middle East, and certain regions of South America with 5% to 40% seropositive prevalence in Japan [8, 9-11]. 

**Figure 7:**
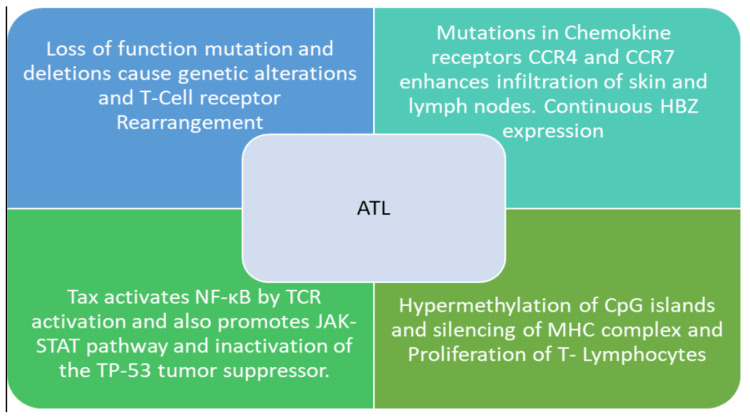
Pathogenesis behind ATL. ATL: Adult T-cell lymphoma; TCR: T-cell receptor; MHC: major histocompatibility complex; JAK-STAT: Janus kinase-signal transducer and activator of transcription

HTLV-1 virus is transmitted through vertical routes (breastfeeding and transplacental transmission), infected blood products (needle sharing, blood, and blood product transfusion), and sexual contact [12]. Viral entry into host cells occurs through various cell signaling mechanisms and cell surface receptors. Interaction between the target cell receptors and viral membrane proteins facilitates viral entry, replication, and dissemination into other host cells. Once the virus enters the target cell, viral RNA encodes the double-stranded viral DNA which incorporates into the host cell genome [13]. HTLV-1 virus genome consists of gag, pol, and env similar to other retroviruses; however, Tax and HBZ (helix- basic- loop zipper) are oncogenic genes implicated in malignant transformation and pathogenesis of ATL [14,15]. Tax, Rex, and HBZ are transcriptional protein factors that regulate the fate of HTLV- 1 virus-infected cells. Tax promotes cellular proliferation through multiple mechanisms including inhibition of apoptosis, upregulation of cytokines, growth factors, and adhesion molecules [16]. Additionally, the Tax-oncoprotein also indirectly promotes inappropriate cellular survival via constitutive activation of the JAK-STAT pathway and inactivation of the TP-53 tumor suppressor [1,3]. While some cells become carriers of the virus and enter a latency period, some target cells undergo clonal expansion that eventually causes ATL [17]. Development of ATL requires multiple genomic and molecular derangements. Figure 7 summarizes the pathogenesis behind ATL. 

HTLV-1 is associated with other diseases in addition to ATL. Some of the other disease entities include tropical spastic paraparesis and inflammatory conditions such as uveitis, myositis, Sjogren’s syndrome, infective dermatitis, and polyneuropathies [8]. Patients with HTLV-1 are immunocompromised and can also develop accelerated infections such as tuberculosis, pneumocystis, and especially Strongyloides [18]. HTLV-1 affects multiple cell lines of the reticuloendothelial system which causes the classic symptoms of ATL. ATL is classified into smoldering, chronic, lymphoma, acute, and extranodal primary cutaneous subtypes [19]. Smoldering and chronic variants have an indolent course while acute, extranodal, and lymphoma subtypes are aggressive forms of the disease with poor prognosis. Table 2 shows the similarities and differences between ATL variants. 

**Table 2:** Similarities and differences between ATL variants. ATL: Adult T-cell lymphoma

	Acute	Chronic	Lymphoma	Smoldering	Extra Nodal
Lymphocytosis	+	+/abnormal t- lymphocytes	-	+/ abnormal t- lymphocytes	-
Lymphadenopathy	+	+	+	-	-
Skin Lesions	+	+/-	+/-	+	++/papules, nodules, and tumors
Hepatosplenomegaly	+	-	-	-	-

HTLV-1 is detected through serology with ELISA or Western blot. Patients with signs and symptoms of ATL should undergo bone marrow or skin lesions biopsy. Peripheral blood smear in ATL shows classic flower cells, which are composed of condensed chromatin, absent nucleoli, and basophilic cytoplasm [12]. Additional findings from laboratory investigations that support the diagnosis include elevated serum LDH and hypercalcemia. Immunophenotype analysis of ATL tumor cells is positive for CD 3, CD 4, CD 25, and CD 30 with of loss of CD 7 [19].

Chemotherapy, antivirals, and allogeneic hematopoietic stem cell transplant are a few treatment strategies implemented over the years. Effective chemotherapy regimens include CHOP (cyclophosphamide, doxorubicin, vincristine, and prednisone) or VCAP-AMP-VECP (vincristine, cyclophosphamide, doxorubicin, and prednisone; doxorubicin, ranimustine, and prednisone; vindesine, etoposide, carboplatin, and prednisone) [12]. However, socioeconomic factors limit the availability of these drugs in many countries. Antivirals such as zidovudine (AZT) and interferon-alpha are used to treat indolent variants of ATL. As with our patients, chronic and smoldering subtypes are also treated with ultraviolet light B for the cutaneous lesions. Stem cell transplant and targeted therapies with monoclonal antibodies directed against CD 25, CCR4, and anti-PD-1 are ongoing but promising clinical trials for aggressive forms of ATL [12]. Recently, anti-CCR4 therapy, mogamulizumab, is approved for treatment in Japan for rapidly progressive ATL [19]. 

Preventive strategies must be employed in endemic countries and also in places with high immigrant populations to abate transmission rates. A few of the approaches include 1. prenatal screening of mothers to curb vertical transmission, 2. screening of blood donors, and 3. safe sex counseling to prevent sexual transmission. Transplant donors from endemic countries and immunosuppressed patients with signs of opportunistic infections must always be screened for the HLTV-1 virus. 

## Conclusions

In summary, ATL is a rare, yet aggressive malignancy caused by HTLV-1 virus. Patients with ATL can have an atypical presentation especially manifesting with skin lesions. Indolent HTLV-1 infection can present with erythroderma as with our patient. It poses a unique challenge for physicians, as it can mimic other inflammatory skin conditions. Therefore, early recognition and treatment are important to improve the outcome of the affected individual to prevent complications and death. Our patient presented with erythroderma initially and based on clinical signs and symptoms underwent the biopsy diagnostic of ATL. Despite aggressive treatment including chemotherapy, stem cell transplantation, and targeted therapies, patients often relapse. Due to the aggressive nature of the disease, long-term remission is challenging, and the survival rate remains low. Further research with monoclonal antibodies is required to control tumor invasion. Clinical trials with combination therapies should also be explored as a promising treatment for this unusual condition. 
